# Remission in psoriatic arthritis: is it possible and how can it be predicted?

**DOI:** 10.1186/ar3021

**Published:** 2010-05-18

**Authors:** Tajvur P Saber, CT Ng, Guillaume Renard, Bernadette M Lynch, Eliza Pontifex, Ceara AE Walsh, Alexia Grier, Marian Molloy, Barry Bresnihan, Oliver FitzGerald, Ursula Fearon, Douglas J Veale

**Affiliations:** 1 Department of Rheumatology, Dublin Academic Medical Centre, St Vincent's University Hospital, Elm Park, Dublin 4, Ireland; 2 The Conway Institute of Biomolecular and Biomedical Research, University College Dublin, Belfield, Dublin 4, Ireland

## Abstract

**Introduction:**

Since remission is now possible in psoriatic arthritis (PsA) we wished to examine remission rates in PsA patients following anti tumour necrosis factor alpha (TNFα) therapy and to examine possible predictors of response.

**Methods:**

Analysis of a prospective patient cohort attending a biologic clinic, between November 2004 and March 2008, was performed prior to commencing therapy and at regular intervals. Baseline clinical characteristics including demographics, previous disease-modifying antirheumatic drug (DMARD) response, tender and swollen joint counts, early morning stiffness, pain visual analogue score, patient global assessment, C reactive protein (CRP) and health assessment questionnaire (HAQ) were collected.

**Results:**

A total of 473 patients (152 PsA; 321 rheumatoid arthritis (RA)) were analyzed. At 12 months remission, defined according to the disease activity score using 28 joint count and CRP (DAS28-CRP), was achieved in 58% of PsA patients compared to 44% of RA patients, significant improvement in outcome measures were noted in both groups (*P *< 0.05). Analysis of a subgroup of PsA and RA patients matched for DAS28-CRP at baseline also showed higher numbers of PsA patients achieving remission. Linear regression analysis identified the HAQ at baseline as the best predictor of remission in PsA patients (*P *< 0.001).

**Conclusions:**

DAS28 remission is possible in PsA patients at one year following anti-TNF therapy, at higher rates than in RA patients and is predicted by baseline HAQ.

## Introduction

Psoriatic arthritis (PsA) is a chronic inflammatory arthritis, usually seronegative for rheumatoid factor associated with psoriasis [1,2]. The clinical phenotype varies widely, which has led to difficulties with classification, diagnosis and therefore predicting prognosis. Initially, PsA was considered a benign disease, one study suggesting only 11% of patients developed erosions over seven years [3]. However, in the same journal it was highlighted that a number of reports suggested a high occurrence of erosions in between 46 to 62% of patients [4]. The incidence of PsA varies from 5.4 to 42% depending on the report. In a Finnish population based study 46% developed erosions [5] and in another study 62% of patients worsened and the pattern of disease changed over time [6]. Several recent studies, however, suggest PsA is progressive, often disabling and associated with an increased mortality [7]. In a study of PsA, in an early arthritis clinic, it accounted for 13% of new patients and progressive, erosive damage occurred in almost 50% patients in the first two years [8].

In the absence of evidence from randomized clinical trials, Methotrexate (MTX) is generally accepted to be useful for the control of peripheral arthritis, but has little efficacy in spinal disease [9]. In a study of early PsA, however, erosive damage appeared to develop even when MTX therapy was commenced early [8]. This raises the question 'Should anti-TNF agents be introduced early?' Remission implies the reversibility of functional impairment, minimal or no progression to joint destruction, and at least a theoretic potential to heal a damaged joint [10]. Recent studies suggest remission may now be attainable in rheumatoid arthritis (RA) with the advent of anti-TNF therapy [11], however RA remission has been defined by different criteria (i) DAS28 value of ≤2.6 [12] (ii) imaging - no progression on X-ray/Ultrasound/MRI; or (iii) American College of Rhuematology (ACR) criteria [13]. Drug-induced remission may be defined as minimal or no clinically detectable disease activity in the presence of continuing drug treatment, which is not stopped or interrupted, but is required to retain the remission state [14]. Drug-free remission persists in the absence of medication. In a recent editorial, de Vlam and Lories highlighted that remission may be a possible goal in PsA [15].

In the current prospective study, we specifically examine clinical and laboratory measures of disease activity to estimate remission rates in PsA patients and examine associated predictive factors.

## Materials and methods

We established a biologic outpatient clinic and prospective database to provide close monitoring and follow-up of patients on biologic therapies. Patients commencing Infliximab, Adalimumab and Etanercept were assessed at baseline, 3, 6 and 12 months with clinical examination, swollen joint count (SJC) and tender joint count (TJC), visual analogue scores (VAS) for pain and for patient global, Health Assessment Questionnaire (HAQ). Erythrocyte sedimentation rate (ESR) and C-reactive protein (CRP) were measured and the 28-joint count Disease Activity Score, DAS28 calculated. RA patients fulfilled diagnostic criteria for according to American College of Rheumatology criteria [13], and PsA patients satisfied validated CASPAR criteria [16]. All patients had clinically active disease, with DAS28 > 3.2 points despite conventional DMARD therapy, and were offered treatment with biologic agents. Patients who had previously received biologic therapy were excluded from this analysis.

Patients received education prior to commencing biologic therapy and thereafter gave fully informed verbal consent. Details of patient age, gender, diagnosis, disease duration, RF and CCP antibody status were collected. DAS28 which has been validated for use in PsA [17] and RA patients and modified (HAQ) [18] was calculated. DAS28 response was analyzed by change from baseline, and by the European League Against Rheumatism (EULAR) criteria response categories [19]. All treatment was fully in compliance withthe Helsinki Declaration and the analysis was approved by the St. Vincent's University Hospital ethics committee.

### Statistics

Statistical analysis was performed using SPSS 16 for Windows. Clinical data are expressed as median values and range unless otherwise stated. Comparisons of improvement within a disease group at different time points were performed using Wilcoxon Rank Sign test. Chi square test for categorical data and Mann-Whitney U test for continuous data were used to evaluate the statistical significance of the difference between the two independent groups, PsA and RA.

## Results

### Demographic characteristics

**Figure 1:**
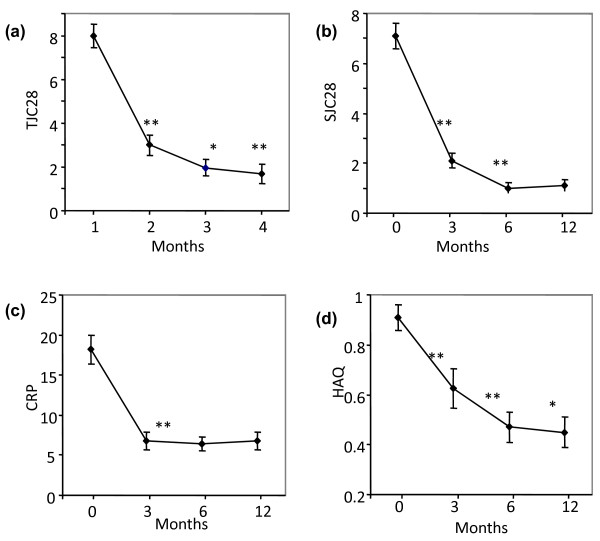
**Individual clinical outcome measures in Psoriatic arthritis over time**. The top left graph depicts the rapid response of Tender Joint Count (TJC) to commencement of treatment with a biologic agent. The top right shows similar response of Swollen Joint Count (SJC). The bottom left graph shows CRP mg/L decline with biologic therapy. The bottom right graph shows HAQ and its dramatic improvement. Time is shown as O for baseline and then 3, 6 and 12 months.

Data were collected and analyzed from a total of 473 patients (152 PsA; 321 RA) over a one-year follow-up period. Baseline describes time of first dose of anti TNF therapy. Baseline characteristics including demographics, clinical and laboratory data are shown in Table 1. These values are expressed as medians (range). As expected from previous cohort studies, the PsA patients tended to be younger, male and had lower joint counts and disease activity scores in comparison to RA patients. PsA patients received Infiximab-13%, Adalimumab-37% and Etanercept-50%, whereas in RA these drugs were used in 6.8%, 56% and 37.2% of patients respectively. The PsA cohort had oligoarticular disease (27.4%) and polyarticular disease (72.6%). Baseline joint counts, CRP and HAQ for PsA patients are shown in Figure 1.

**Table 1:** Baseline variables shown in median(range) unless otherwise stated

	PsA (n = 152)	RA (n = 321)	*P *value
Age	45(15 to 77)	56(17 to 85)	<0.001
Male	47.7%	29.1%	<0.001
Female	52.3%	70.9%	
Disease Duration	8(0.1 to 45)	10(0.1 to 42)	= 0.01
Previous MTX Use	94.6%	92.6%	
Rheumatoid Factor Positive	0.0%	77.6%	
Anti CCP Positive	0%	72%	
VAS (mm)	60(10 to 100)	60(10 to 100)	ns
Concomitant DMARD	36%	68.4%	
Steroid use	14%	41.5%	<0.001

DMARD: disease modifying agents of rheumatic diseases; MTX: methotrexate;

PsA: psoriatic arthritis; RA: rheumatoid arthritis; VAS: visual analogue scale of patient global health

### Clinical outcome measures in PsA patients

In PsA patients the TJC was 7.9 ± 0.532 (mean ± SEM) at baseline, reduced to 3 ± 0.477 at 3 months, 1.97 ± 0.38 at 6 months and 1.79 ± 0.45 at 12 months (*P *< 0.01) (Figure 1A). The baseline SJC was 7.13 ± 0.51, reduced to 2.16 ± 0.3 at 3 months, 1 ± 0.22 at 6 months and 1 ± 0.25 at 12 months (*P *< 0.01) (Figure 1B). The patient global VAS was 5.5 ± 0.19 at baseline and reduced to 2.9 ± 0.22 at 3 months had a value of 2.3 ± 0.23 at 6 months and then remained at this level so that the mean value at 12 months was 2.2 ± 0.25. The baseline CRP in the PsA patients was 18 ± 1.83 which fell to 6.8 ± 1.14 at 3 months (*P *< 0.01) and remained at this level at 6 and 12 months (Figure 1C). The HAQ at baseline was 0.91 ± 0.05 and significantly improved to 0.625 ± 0.08 at 3 months then to 0.470 ± 0.06 at 6 months and maintained out to 12 months (all *P *values < 0.01) (Figure 1D).

### Remission rates in PsA

**Figure 2:**
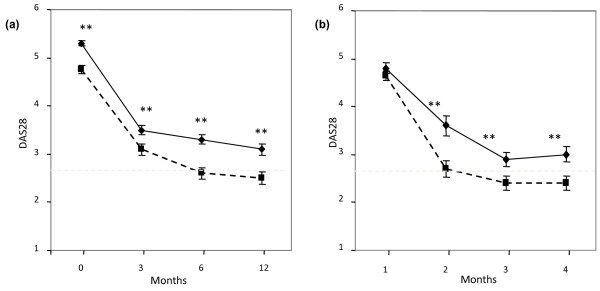
**Comparison of DAS28-CRP response in PsA versus RA patients over time**. **2a **DAS28-CRP in PsA (dotted line) is compared to RA (solid black line). Remission represented by the line at DAS28 value of 2.6. There is a highly significant response of PsA compared to RA at all time points. **2b **shows a subgroup of patients matched for disease activity at baseline (n = 41 in each group) and similar dramatic response.

In the PsA patients the DAS28 remission rate, computed with four variables using the CRP, at 12 months was 58%. This represented a significant change from a baseline of 4.75 ± 0.09 to 3.1 ± 0.12 at 3 and 2.6 ± 0.11 at 6 months, and a further reduction to 2.5 ± 0.13 at 12 months (all *P *values < 0.01) (Figure 2A). A significant improvement in DAS28 was also demonstrated in RA patients from baseline to 12 months, although the overall remission rate of 44% was significantly lower than in the PsA patients. (Figure 2).

**Figure 3:**
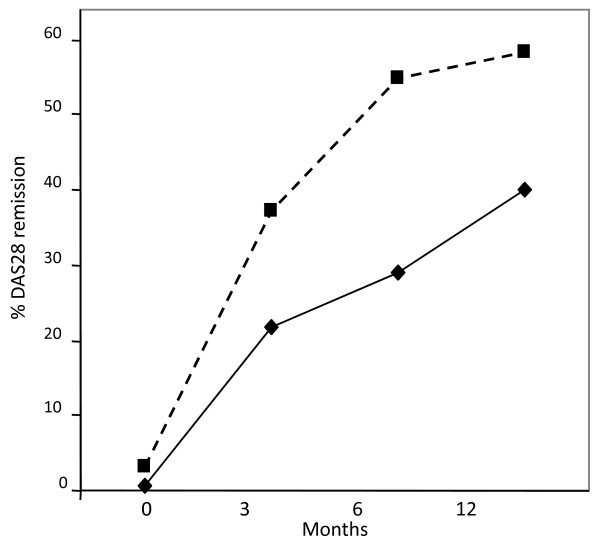
**Percentage of patients in DAS28-CRP remission over time**. PsA represented by the dotted line shows a dramatic immediate response to Biologic therapy compared to RA, represented by solid black line. A total of 58% PsA patients are in remission at 12 months compared to 44% of RA patients.

As there was a significant difference in DAS28 scores between the PsA and RA at baseline, we analysed a subgroup of PsA (n = 41) and RA (n = 41) patients which were matched for baseline DAS28 scores (Figure 2B). Analysis of these matched PsA and RA patient subgroups still demonstrated a significantly higher number of PsA patients attaining remission 63.5% at 12 months compared to the RA group 41.4% (Figure 2B). In addition, the rate of achieving remission in the PsA patients was significantly higher compared to RA patients at 3, and 6 and 12 months (all *P *< 0.01) (Figure 3).

### Predictors of remission

In an individual analysis of baseline variables (Table 2) a number of features appeared to predict the clinical outcome of remission as defined by DAS28 < 2.6. Specifically, in PsA patients male gender, HAQ, Patient global VAS and early morning stiffness were independently associated with increased remission. Linear regression analysis of baseline characteristics, however suggest that the HAQ at baseline is the sole predictor of DAS28 at one year (*P *< 0.001).

**Table 2:** DAS28 at one year prediction by correlation with individual factors at baseline

	*P *value
HAQ	<0.001
Gender	0.004
Pt global VAS	0.035
Stiffness	<0.001
Pain	0.032
Fatigue	0.06

HAQ: health assessment questionnaire; Pt global VAS: patient global visual analogue scale of disease activity.

## Discussion

In this study, we show a significant response to anti-TNF therapy in routine clinical practise with DAS28 remission in 58% PsA patients compared to 44% of RA patients. There were significant differences in single variables and the DAS28 scores between PsA and RA patients at baseline. This may reflect differences in pattern of joint involvement and/or lower CRP levels frequently noted in PsA compared with RA patients [17]. To reduce the possible bias due to differences at baseline between PsA and RA, we analysed a subgroup matched for baseline DAS28, this still showed a significantly greater response in PsA patients compared to RA. While response rates in PsA have been looked at before, these studies looked into EULAR response rates [20] and did not comment on DAS28 response, or remission, as we do here.

Individual variables including tender and swollen joint counts, CRP, patient global VAS and the HAQ showed significant improvement in both patient groups, most parameters showed the greatest response within the first 3 months, however significant improvements were seen between 3 and 12 months, residual tender and swollen joints were more common in RA patients. The mean CRP fell in both PsA and RA patients, it fell more sharply in the PsA group overall, however, in the matched PsA and RA subgroup analysis CRP changes were comparable. The patient reported outcomes such as patient global VAS, pain VAS and HAQ also showed significant improvement in both patient groups. PsA patients had less tender and swollen joints and stiffness at 12 months, but also a significantly lower DAS 28 and HAQ.

Fifty-eight percent of PsA patients were in DAS28 remission at 12 months compared to 44% of RA patients. When the two groups were analysed as regards EULAR good response, that is, a DAS28 of <3.2 at endpoint and and improvement of more than or equal to 1.2, then 73% of PsA, but only 51% of RA patients, met this criteria. It was interesting to note that male PsA patients attained significantly lower DAS28 scores than females 1.88 vs 2.65 at one year (*P *< 0.05). Since the initial DAS28 in PsA was lower than RA patients when biologic treatment started we compared remission rates in PsA vs RA, by analyzing a subset of patients matched for disease activity at baseline (n = 41, in each group). Remission rates defined by DAS28 were significantly greater in PsA patients (*P *= 0.01), 63.5% PsA patients vs 41.4% RA patients at 12 months even in this particular group.

This is the first study to examine remission rates in a cohort of PsA patients from routine clinical practise studied prospectively in a dedicated biologic clinic. The finding of DAS28 remission in almost two-thirds of patients is significantly higher than the observed level in RA patients, even when matched for baseline disease activity. Remission has become an attainable goal in the treatment of RA, the recent Combination of Methotrexate and etanercept in Active Early Rheumatoid (COMET) study suggesting high remission rates in RA patients with high levels of disease activity commenced on treatment early [21]. The aim of remission in the treatment of RA has been adopted as desirable and feasible by EULAR in the most recent recommendations [22]. In a recent editorial, the potential of remission in PsA was considered to be a realistic goal [15]. There are several possible criteria which may be used in the definition of remission, including single clinical variables, response criteria, pooled indices such as the DAS and ACR and then imaging technology criteria. In an important study of the performance of response criteria for assessment of peripheral arthritis in PsA, Fransen et al, compared a detailed analysis of individual items and pooled indices in dicriminating change in two clinical trial data sets [19]. The authors concluded that response criteria and pooled indices, specifically the EULAR response criteria, performed better than the ACR or the PsARC in discriminating active from placebo drugs. In addition, they found that the DAS and DAS28 performed better than single core-set measures in PsA. Furthermore, two studies have reported that the DAS28 is a valid instrument for measuring disease activity with respect to response to biologic therapies [19,23]. In measuring remission in this prospective cohort study we have applied the DAS28, as a validated measure of disease activity in PsA patients treated with biologics, and in the knowledge that such a pooled index developed for RA has been shown to be useful for assessment of the peripheral arthritis of PsA. The results of this study suggest therefore that, using a single measure of disease activity with an agreed level defined as remission, biologic therapies result in high remission rates in PsA patients, greater than a comparator group of RA patients even when matched for baseline disease activity.

In this study we found a number of individual baseline parameters were associated with remission examining independent correlations. In particular, in PsA patients, male gender and the patient-derived indices including HAQ, patient global VAS and early morning stiffness appeared to be associated with remission. Linear regression analysis however failed to confirm all these variables as predictors of remission and suggested the association was strongest between baseline HAQ and remission. A previous report of PsA patients treated with anti-TNF agents had identified improvement in the DAS28 score as the best predictor of improvement in quality of life (QoL) measured using the SF36 [24]. Interestingly, the authors of this paper also found significantly higher QoL responses in their PsA cohort compared to the RA cohort studied, a result which confirmed an earlier report from the Norwegian registry [25]. In a recent international multicenter study of RA patients [26], measures of HAQ also behaved differently in men and women. We therefore reanalysed the data in our RA cohort and found there are differences in men and women in relation to HAQ response; however, the size of the response in male PsA and RA patients is similar. The association of male gender with remission was unexpected and raises an intriguing question: Why do male PsA patients show a better response to therapy? One possible explanation is related to testosterone levels, which have previsouly been reported to be higher in HLAB27 positive subjects [27], and may have a protective effect in seronegative spondyloarthopathies [28]. Indeed, it is intriguing to hypothesize that testosterone levels may augment the response of PsA patients to biologic therapies.

## Conclusions

This is the first prospective study of biologic therapy in a routine clinical inflammatory arthritis cohort to demonstrate a remission rate of over 58% in patients with PsA. Remission, defined by DAS28, has been validated in PsA biologic therapy trials, and shown to be more responsive than single outcome measures. Furthermore, we have shown that DAS remission is significantly higher in this PsA cohort compared to an RA cohort, even when matched for baseline disease activity. The remission response in the PsA patients appears to be most strongly associated with the patient-derived outcome measure of function - HAQ. These data suggest that remission is both a realistic and achievable goal in the majority of PsA patients.

## Abbreviations

ACR: American College of Rhuematology; Anti TNFα: anti tumour necrosis factor alpha; CRP: C reactive protein; DMARD: disease modifying agents of rheumatic diseases; ESR: erythrocyte sedimentation rate; EULAR: European League Against Rheumatism; HAQ: health assessment questionaire; MRI: magnetic resonance imaging; MTX: methotrexate; PsA: psoriatic arthritis; QoL: quality of life; RA: rheumatoid arthritis; SJC: swollen joint count; TJC: tender joint count; VAS: visual analogue scale.

## Competing interests

TS has received a Newman scholarship through UCD supported by Centocor Ltd and so on. OF has grant/research support from Abbott and BMS; he also acts as a consultant for Abbott and UCB and is on the Speakers Bureau for Abbott. DJV has grant/research support, acts as a consultant for and is on the Speakers Bureau for from Abbott, GSK, Centocor, Wyeth, Pfizer and Schering Plough. The other authors declare that they have no competing interests.

## Authors' contributions

TPS was responsible for author conception, design, acquisition of data, analysis and interpretation of data, and also for drafting of the manuscript. VCTN was responsible for conception, acquisition of data, analysis and interpretation of data. GR was responsible for analysis and interpretation of data, and also for drafting of the manuscript. BML, EP, CAEW, AG and MM were responsible for acquisition of data. BB was responsible for conception and interpretation of data, and, along with OF, was responsible for conception and acquisition of data. UF was responsible for the design, analysis and interpretation of data, and also for drafting of the manuscript. DJV was responsible for conception, design, acquisition of data, analysis and interpretation of data and drafting of the manuscript.
